# Quantification of task-dependent cortical activation evoked by robotic continuous wrist joint manipulation in chronic hemiparetic stroke

**DOI:** 10.1186/s12984-017-0240-3

**Published:** 2017-04-17

**Authors:** Martijn P. Vlaar, Teodoro Solis-Escalante, Julius P. A. Dewald, Erwin E. H. van Wegen, Alfred C. Schouten, Gert Kwakkel, Frans C. T. van der Helm, on behalf of the 4D-EEG consortium, Jan de Munck, Carel Meskers, Mique Saes, Luuk Haring, Caroline Winters, Aukje Andringa, Dirk Hoevenaars, Ines de Castro Fernandes, Sarah Zandvliet, Andreas Daffertshofer, Jun Yao, Yuan Yang, Mark van de Ruit, Konstantina Kalogianni, Lena Filatova

**Affiliations:** 1 BioMechanical Engineering Department, Faculty of Mechanical, Maritime and Materials Engineering, Delft University of Technology, Delft, The Netherlands; 2 Department of Physical Therapy and Human Movement Sciences, Feinberg School of Medicine, Northwestern University, Chicago, IL USA; 3 Department of Biomedical Engineering, McCormick School of School of Engineering, Northwestern University, Evanston, IL USA; 4 MIRA Institute for Biomedical Technology and Technical Medicine, Laboratory of BioMechanical Engineering, University of Twente, Enschede, The Netherlands; 5 VU University Medical Centre, Amsterdam Neurosciences, Amsterdam, The Netherlands; 6 MOVE Research Institute Amsterdam, Amsterdam, The Netherlands

**Keywords:** Stroke, Electroencephalogram, Robotic joint manipulation, Sensory impairment, Somatosensory system, Steady-state evoked response

## Abstract

**Background:**

Cortical damage after stroke can drastically impair sensory and motor function of the upper limb, affecting the execution of activities of daily living and quality of life. Motor impairment after stroke has been thoroughly studied, however sensory impairment and its relation to movement control has received less attention. Integrity of the somatosensory system is essential for feedback control of human movement, and compromised integrity due to stroke has been linked to sensory impairment.

**Methods:**

The goal of this study is to assess the integrity of the somatosensory system in individuals with chronic hemiparetic stroke with different levels of sensory impairment, through a combination of robotic joint manipulation and high-density electroencephalogram (EEG). A robotic wrist manipulator applied continuous periodic disturbances to the affected limb, providing somatosensory (proprioceptive and tactile) stimulation while challenging task execution. The integrity of the somatosensory system was evaluated during passive and active tasks, defined as ‘relaxed wrist’ and ‘maintaining 20% maximum wrist flexion’, respectively. The evoked cortical responses in the EEG were quantified using the power in the averaged responses and their signal-to-noise ratio.

**Results:**

Thirty individuals with chronic hemiparetic stroke and ten unimpaired individuals without stroke participated in this study. Participants with stroke were classified as having severe, mild, or no sensory impairment, based on the Erasmus modification of the Nottingham Sensory Assessment. Under passive conditions, wrist manipulation resulted in contralateral cortical responses in unimpaired and chronic stroke participants with mild and no sensory impairment. In participants with severe sensory impairment the cortical responses were strongly reduced in amplitude, which related to anatomical damage. Under active conditions, participants with mild sensory impairment showed reduced responses compared to the passive condition, whereas unimpaired and chronic stroke participants without sensory impairment did not show this reduction.

**Conclusions:**

Robotic continuous joint manipulation allows studying somatosensory cortical evoked responses during the execution of meaningful upper limb control tasks. Using such an approach it is possible to quantitatively assess the integrity of sensory pathways; in the context of movement control this provides additional information required to develop more effective neurorehabilitation therapies.

## Background

The cerebral cortex plays an important role in feedforward (i.e. voluntary motor drive) and feedback control (i.e. reflexes and modulation of spinal reflexes) of human movement [1]. Cortical damage after stroke impairs both feedforward and feedback control. Altered feedforward control after stroke has been thoroughly studied and may lead to motor impairments such as weakness and abnormal synergy-dependent motor control [2, 3].

Cortical involvement in feedback control (including sensorimotor integration and spinal reflex modulation) requires connectivity between somatosensory receptors in the periphery and the sensorimotor cortex, yet compromised integrity of this somatosensory system after stroke has received little attention in the literature. Understanding the impact of sensory impairment, as well as motor impairment, is highly relevant for the development and selection of neurorehabilitation therapies aimed to enhance and normalize motor control [4–7] and for evaluating their effectiveness.

Proprioceptive and tactile information are required for feedback control of a joint, and can be studied in an experimental setting by disturbing the joint via a robotic manipulator during motor control tasks. This robotic joint manipulation results in activation of spinal reflex loops [8–10] as well as in activation of the somatosensory cortex via high-resolution sensory pathways [11]. However, the cortical activity evoked by joint manipulation and consequently the cortical involvement in feedback control have received less attention.

In able-bodied individuals, evoked cortical responses to robotic joint manipulation have been studied with transient [12, 13] and continuous disturbances [14–16]. Continuous disturbances uninterruptedly provide input to the sensory system, allowing for studying movement control and somatosensory cortical activity during meaningful motor tasks. This study determines the cortical representation of afferent (proprioceptive and tactile) information in individuals with chronic hemiparetic stroke under different upper limb control conditions, relying on objective metrics derived from the electroencephalogram (EEG). Here, the goal is to quantify evoked cortical activation in individuals with chronic hemiparetic stroke, through a combination of robotic continuous joint manipulation of the paretic limb and high-density EEG. The evoked cortical activation reveals the integrity of the connections between sensory receptors in the periphery and the sensorimotor cortices.

It is hypothesized that, due to stroke-induced damage to the somatosensory system, individuals with clinically assessed proprioceptive and tactile impairment will show decreased cortical evoked responses to continuous joint manipulation in the absence of voluntary motor activity of the affected upper limb, as compared to unimpaired persons. In general, when voluntary motor activity of the affected upper limb is required, individuals with hemiparesis have been shown to recruit their contralesional brain hemisphere, i.e. ipsilateral to the movement [17–20]. It is unclear, however, what this recruitment means with regard to somatosensory (i.e. afferent) evoked cortical activity, as the anatomical pathways conducting proprioceptive and tactile information mainly connect to the contralateral hemisphere [21]; thus, increased evoked cortical activation of the ipsilateral hemisphere is not expected.

## Methods

### Participants

Thirty participants with chronic hemiparetic stroke (i.e. at least 6 months post stroke, with initial hemiparesis) participated in this study (12 female, average age 64 years, SD = 11, see Table 1). The inclusion criteria were (i) first-ever ischemic stroke in an area supplied by the anterior, medial, and/or posterior cerebral arteries, (ii) age ≥ 18 years, (iii) no severe cognitive deficits (mini mental state examination score of ≥19), and (iv) able to sit in a wheelchair for at least 2 h. Exclusion criteria were previously existing pathological neurological conditions, pacemaker or other metallic implants, previously existing orthopedic limitations of the upper limb that would affect the results, and botulinum-toxin injections or medication that may influence upper limb function in past 3 months. Additionally, ten unimpaired age-matched volunteers without stroke were recruited as control group (3 female, average age 59 years, SD = 9). The inclusion (ii–iv) and exclusion criteria for the unimpaired volunteers were the same as for the participants with stroke. All participants gave written informed consent prior the experiments. The study has been approved by the Medical Ethics Reviewing Committee of the VU Medical Center, Amsterdam (protocol number 2014.140, Dutch Central Committee on Research Involving Human Subjects, CCMO, protocol number NL47079.029.14). This study was conducted in accordance with The Declaration of Helsinki.

**Table 1:** Participants with stroke grouped by level of sensory impairment (sub-sorted by FMA-UE score)

ID	Sensory group	EmNSA					FMA-UE (max 66)	Months post stroke	Age (yr)	Gender M:male F:female	Affected side	Handed-ness
		LT	P	PP	D	PR						
1	severe	0	1	1	N/A	1	6	6	71	F	L	R
2	severe	0	1	1	N/A	1	8	21	54	M	L	L
3	severe	1	1	1	N/A	0	9	212	66	F	R	R
4	severe	1	1	1	0	1	10	6	64	M	R	R
5	severe	1	1	1	N/A	1	20	142	68	M	L	L
6	severe	1	1	1	N/A	1	26	15	72	M	L	L
7	severe	1	2	2	1	1	62	7	77	M	L	L
8	mild	1	2	2	1	2	9	71	59	M	L	L
9	mild	1	2	2	1	2	10	81	48	M	L	L
10	mild	2	2	2	1	2	10	6	93	F	R	R
11	mild	2	2	2	1	2	54	26	67	M	R	R
12	mild	2	2	2	1	2	56	11	56	M	L	L
13	mild	1	2	2	1	2	59	53	50	F	R	R
14	mild	2	2	2	1	2	60	11	61	F	R	R
15	mild	2	2	2	1	2	63	35	76	F	L	L
16	mild	2	2	2	1	2	63	10	78	F	R	R
17	mild	2	2	2	1	2	64	23	65	M	L	L
18	mild	2	2	2	1	2	64	6	70	F	R	R
19	mild	2	2	2	1	2	64	6	75	F	L	R
20	none	2	2	2	2	2	11	6	52	F	R	R
21	none	2	2	2	2	2	13	82	64	M	L	L
22	none	2	2	2	2	2	20	6	77	M	L	R
23	none	2	2	2	2	2	39	50	62	M	R	R
24	none	2	2	2	2	2	48	35	50	M	R	R
25	none	2	2	2	2	2	58	75	55	M	L	L
26	none	2	2	2	2	2	59	41	49	F	L	L
27	none	2	2	2	2	2	60	6	73	M	L	R
28	none	2	2	2	2	2	66	67	68	F	R	R
29	none	2	2	2	2	2	66	10	57	M	L	L
30	none	2	2	2	2	2	66	88	48	M	R	R

Number of participants in sensory impairment groups: severe (6), mild (13), none (11). Subscores for EmNSA-UE (2: no impairment, 1: some impairment 0: fully impaired) LT:light touch, P:pressure, PP:pinprick, D:discrimination, PR:proprioception. N/A means this test was not performed due to tactile impairment as established in LT, P and PP

The levels of sensory and motor impairment of each participant with chronic stroke were assessed using the Erasmus modification of the Nottingham Sensory Assessment for the upper extremity (EmNSA-UE) [7] and the Fugl-Meyer Assessment for the upper extremity (FMA-UE) [22], respectively. Participants with stroke were classified in three groups according to their level of sensory impairment in a similar way as in Stolk-Hornsveld, et al. [7]. Participants who achieved a full score on each subtest of the EmNSA-UE were classified as having no sensory impairment. Participants with a reduced score in one or two subtests were classified as having mild sensory impairment, whereas participants with a reduced score on more than two subtests of the EmNSA-UE were classified as having severe sensory impairment.

### Experimental protocol

Processing and integration of sensory information was evaluated with a passive and an active upper limb control task. In this protocol a robotic manipulator applied continuous periodic disturbances to the wrist to provide sensory stimulation and to challenge task execution. This protocol focuses on the paretic forearm acknowledging that the upper limb is often more severely affected and return of some dexterity is essential for activities of daily living [23–25].

#### Experimental setup

**Fig. 1:**
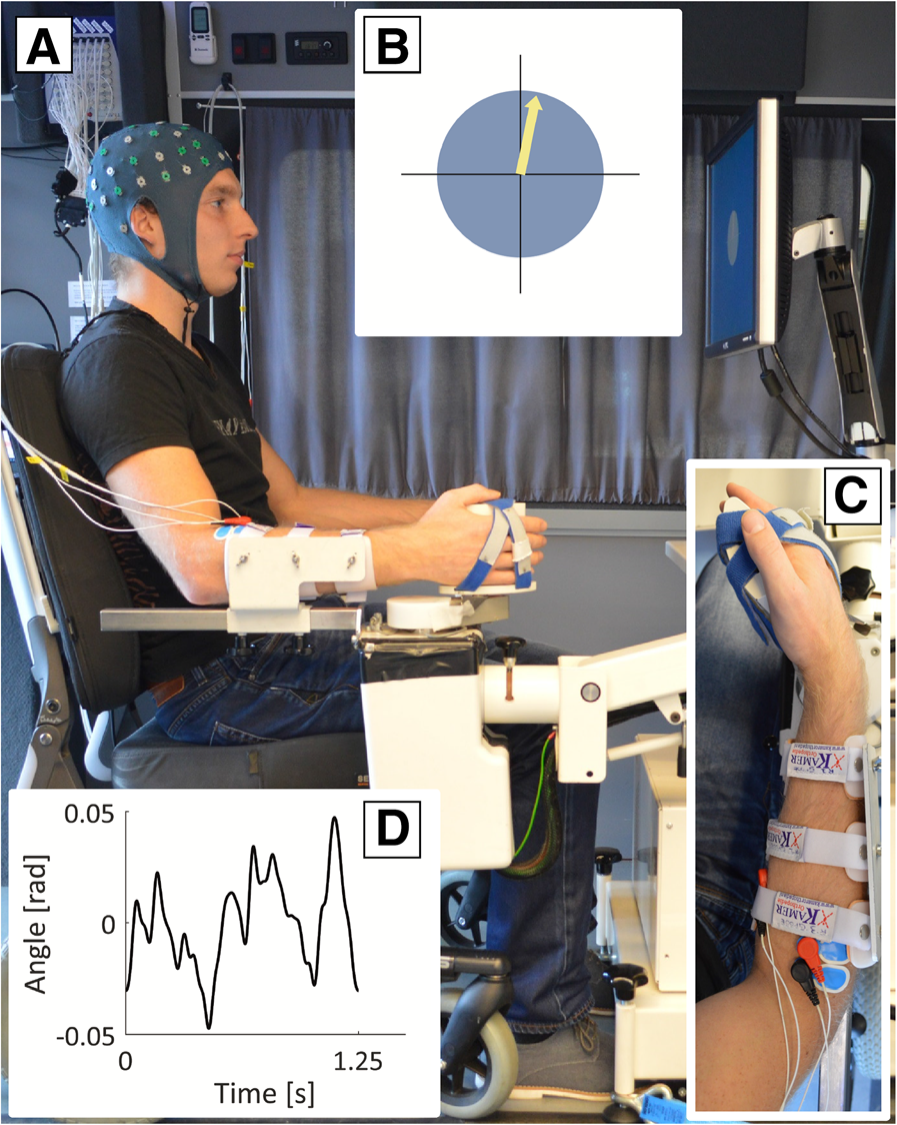
Experimental setup. **a** The forearm of the participant is strapped into an armrest and the hand is strapped to the handle of the robotic manipulator, requiring no hand force to hold the handle. **b** Visual feedback as presented to the participant. The circle and crosshairs are always visible. The yellow arrow is only visible during the active task and points up if the target torque is applied. **c** Close-up of the arm in the robotic manipulator. The wrist joint is aligned with the axis of the motor and is placed in the neutral angle, defined as 20° wrist flexion. **d** One period of the disturbance signal applied to the wrist (root-mean-square of 0.02 rad). Zero radians corresponds to the neutral angle of the wrist

All EEG recordings were performed in a customized measurement van (Volkswagen Crafter, Wolfsburg, Germany) equipped with stabilizing feet, shaded windows, curtains, a wheelchair (Ibis, Sunrise Medical Incorporated, Fresno, CA, USA), and all experimental equipment (see Fig. 1a). The participant’s stimulated forearm (i.e. paretic arm for participants with stroke or dominant arm for unimpaired participants) was attached to the robotic manipulator (Wristalyzer, MOOG, Nieuw-Vennep, The Netherlands). The wrist joint was aligned to the motor axis and the hand was strapped to the handle of the robotic manipulator using Velcro straps, requiring no active grip from the participant. The shape of the handle ensured forces were applied to the palmar surface of the hand and prevented fingertips from holding the edge. Both tasks were performed with the wrist positioned in a neutral angle, defined as 20° wrist flexion (see Fig. 1c), which allowed for comparison between tasks and participants. A computer screen showed a circle during all tasks, with an arrow that presented task relevant feedback during the active task, as explained below. All visual feedback signals were low-pass filtered (cut-off frequency of 0.6 Hz) to prevent correlation between eye movement and the disturbance signal.

Structural magnetic resonance images of each participant were obtained at the VU Medical Center, Amsterdam, using a Discovery MR750 3 T scanner (GE, Waukesha, WI, USA). T1-weighted volumes were acquired with a 3D fast spoiled gradient-recalled-echo sequence, consisting of 172 sagital slices (256 × 256), using the following acquisition parameters: TR = 8.208 ms, TE = 3.22 ms, inversion time = 450 ms, flip angle = 12°, voxel size 1 × 0.94 × 0.94 mm.

#### Recording system

All signals were recorded using a Refa amplifier (TMSi, Oldenzaal, The Netherlands) sampling at 2048 Hz and without hardware filters (only anti-aliasing). Scalp potentials were recorded using an electrode cap with 64 Ag/AgCl electrodes (TMSi), arranged according to a subset of the extended 10/20 system. A separate electrode (Blue Sensor N, Ambu, Ballerup, Denmark) was connected to the left mastoid process and served as the participant ground. Muscle activity was recorded from two muscles in each forearm (m. flexor carpi radialis and m. extensor carpi radialis brevis) using pairs of unipolar electrodes (Blue Sensor N, Ambu). Signals from the robotic manipulator (recorded and commanded angle and torque) were recorded via optical isolation amplifiers (TMSi) to ensure participant safety.

#### Upper limb control tasks

Passive tasks require no active involvement of the participant, allow for assessment of connectivity between the periphery and the sensorimotor cortex, and are feasible for individuals with severe motor impairment (FMA-UE score lower than 40). Active tasks engage the sensorimotor system in movement control, therewith requiring motor activity and sensorimotor integration. Hence, contrary to passive tasks, active tasks require sensory information for adequate task execution. The active task was included to investigate if voluntary motor drive would be accompanied by an abnormal lateralization of sensory-related cortical activity. This task was chosen such that individuals who suffered a stroke and are capable of some wrist flexion can perform it.

##### Passive task – relaxed wrist

In this task, participants were instructed to relax their wrist and ignore the applied disturbances. A screen in front of the participants showed a static image without any task-related feedback. The periodic angular disturbances applied by the robotic manipulator elicit sustained oscillatory responses in the EEG commonly referred to as steady-state responses (SSR) [26, 27]. In unimpaired persons, the SSR obtained under the passive condition appear in the contralateral sensorimotor cortices.

##### Active task – isotonic wrist torque

In this task participants were instructed to maintain a wrist flexion torque of 20% of the maximum voluntary contraction (MVC), for which they received visual feedback (see Fig. 1b). During this task the participants received the same angular disturbances as in the passive task. The active task was not performed if the participant was not capable of voluntary wrist flexion. Due to limitations of the robotic manipulator, the maximum torque level for the active task was set to 4 Nm. There were three unimpaired participants for whom 20% MVC was higher than 4 Nm (i.e. 5.7 Nm, 5.6 Nm and 4.5 Nm). Additionally, two participants with chronic stroke executed the active task at a higher level than 20% MVC, as the task was not challenging for them at 20% MVC (ID 10, MVC was 1.8 Nm, active task performed at 40% MVC or 0.7 Nm; and ID 20, MVC was 2.8 Nm, active task performed at 50% MVC or 1.4 Nm).

The passive task was performed before the active task. To prevent fatigue in the active tasks, a trial lasted only 12.5 s. For each task 20 trials were recorded. There was a short break between trials which was at least 5 s, or longer if the participant or experimenter deemed necessary. Recording of the active task was stopped in case of severe fatigue or discomfort.

MVC during wrist flexion was determined for the stimulated arm. Participants were verbally encouraged to perform wrist flexion with maximal effort. For participants with wrist flexion torque lower than 5 Nm (experimentally established), the MVC was measured using the robotic manipulator, which maintained a fixed angle (neutral angle). Stronger participants performed this MVC test by exerting flexion torque on a handheld force transducer (MicroFet, Draper, UT, USA). The hand was attached to the robotic manipulator and the neutral angle was approximately maintained.

#### Disturbance signal design

During both the passive and active task the robotic manipulator applied the same continuous periodic angular disturbance signal to the wrist. The disturbance signal was a random-phase multisine signal (e.g. the sum of several sinusoids, each with a random phase) [28], which was designed to stimulate the sensory system in a frequency range relevant to movement control. Control of the wrist at high frequencies is limited by inertia of the limb and by the ability of the muscle to contract at high rates. To accommodate low frequencies the period of the disturbance signal was set to 1.25 s (i.e. frequency resolution of 0.8 Hz). This selection is a tradeoff between frequency resolution and number of periods that can be recorded in a given measurement time, where recording more periods allows for better estimation of an average response. The included sinusoids in the multisine signal were: 0.8, 1.6, 2.4, 3.2, 4.0, 4.8, 5.6, 6.4, 8.0, 9.6, 11.2, 13.6, 16.0, and 19.2 Hz. The frequencies below the natural frequency of the wrist (approximately 3 to 5 Hz for a relaxed wrist) had the highest amplitudes, since in the low frequency region reflexes are most effective due to the inherent time delay associated with them. Frequencies above 4 Hz had decreasing amplitudes (-20 dB/dec). The reason for this is twofold. Firstly, due to inertial properties of the wrist, the forces required to manipulate the wrist increase quadratically with increasing frequency for frequencies above the natural frequency, surpassing the capabilities of the robotic manipulator. Secondly, the muscle spindles serving the Ia afferents are particularly sensitive to velocity information [21, 29].

The angular disturbances were identical for all participants, were always applied in the neutral angle, and had an excursion of 0.02 radians root mean square (see Fig. 1d). The disturbance signal was flipped for recordings on the left hand to have similar flexor/extensor stimulation as in right handed participants. Each trial consisted of ten consecutive periods of the disturbance signal.

### Data processing

All data was processed using MATLAB 8.1 (The Mathworks, Inc., Natick, MA, USA). Topographic representations were generated using EEGLAB [30].

#### Pre-processing

Recorded EEG trials were band-pass filtered between 0.8 Hz and 120 Hz and band-stop filtered in narrow bands around 50 and 100 Hz (line noise and its harmonic). Data were filtered using 4^th^ order Butterworth filters applied bi-directionally to achieve zero-phase filtering. EEG electrodes with high impedance (automatically detected by the recording equipment) were excluded from further analysis. The remaining EEG channels were re-referenced to the common average.

#### Period rejection

After filtering, the trials (12.5 s) were split up into ten periods (1.25 s), according to the period of the disturbance signal. The first two periods were discarded to reduce the influence of transient effects, resulting in a total of 160 periods for each task. Periods were rejected from the active task if the mean wrist torque in the period was not within ±50% of the target torque. If there were less than 80 successfully recorded periods in the active task, the task was excluded from analysis.

#### EEG analysis

##### Independent component analysis

To separate brain signal from artifacts, an independent component analysis (ICA) was performed using the Infomax algorithm [31, 32] as implemented in CUDAICA [33]. ICA was performed on the EEG data of both upper limb control tasks combined, with subsequent rejection of independent components (ICs) corresponding to non-brain signals. ICs representing muscle activity were detected based on an increase of power in the power spectrum for increasing frequency. Components related to blinking and eye movement were detected based on their topographical representation, as well as time course of each component. ICs representing contributions mainly from one electrode were removed. Remaining components were projected back to the electrode level.

##### Outcome metrics

Processing of afferent information was analyzed using the steady-state response (SSR), obtained for each electrode by averaging the responses to all periods:

1 $$ \widehat{\mathit{\mathsf{x}}}\left(\mathit{\mathsf{k}}\right)=\frac{\mathsf{1}}{\mathit{\mathsf{P}}}{\displaystyle \sum_{\mathit{\mathsf{p}}=\mathsf{1}}^{\mathit{\mathsf{P}}}{\mathit{\mathsf{x}}}^{\left[\mathit{\mathsf{p}}\right]}\left(\mathit{\mathsf{k}}\right),} $$

where, $\widehat{\mathit{\mathsf{x}}}$ is the SSR, $\mathit{\mathsf{x}}$ is the recorded signal from one electrode, *k* is a sample in a period $\mathit{\mathsf{p}}$, and $\mathit{\mathsf{P}}$ is the total number of recorded periods. As the recorded EEG signals are electrical potentials measured on the scalp, the magnitude of the signal can easily vary across participants, for example due to differences in skull and scalp conductivity. Therefore, to enable comparison across participants the signal-to-noise ratio (SNR) was used. The SNR was calculated for each electrode by dividing the power in the SSR by the variance across recorded periods:

2 $$ \mathit{\mathsf{S}}\mathit{\mathsf{N}}\mathit{\mathsf{R}}=\frac{{\widehat{\mathit{\mathsf{E}}}}_{\mathit{\mathsf{x}}}}{{\widehat{\sigma}}_{\mathit{\mathsf{x}}}^{\mathsf{2}}}=\frac{{\displaystyle \sum_{\mathit{\mathsf{k}}=\mathsf{1}}^{\mathit{\mathsf{N}}}\widehat{\mathit{\mathsf{x}}}{\left(\mathit{\mathsf{k}}\right)}^{\mathsf{2}}}}{{\displaystyle \sum_{\mathit{\mathsf{k}}=\mathsf{1}}^{\mathit{\mathsf{N}}}\frac{\mathsf{1}}{\mathit{\mathsf{P}}-\mathsf{1}}{\displaystyle \sum_{\mathit{\mathsf{p}}=\mathsf{1}}^{\mathit{\mathsf{P}}}{\left({\mathit{\mathsf{x}}}^{\left[\mathit{\mathsf{p}}\right]}\left(\mathit{\mathsf{k}}\right)-\widehat{\mathit{\mathsf{x}}}\left(\mathit{\mathsf{k}}\right)\right)}^{\mathsf{2}}}}}. $$

Due to the applied filtering and rejection of components representing artifacts, the major cause of variance across periods is expected to be background cortical activity, which is uncorrelated to the periodic disturbance signal.

The difference in power in the SSR between the passive and the active task is calculated to see the intra-participant effect of the active task on the SSR power:

3 $$ \varDelta \mathit{\mathsf{E}}=\frac{{\widehat{\mathit{\mathsf{E}}}}_{\mathit{\mathsf{x}},\mathit{\mathsf{active}}}-{\widehat{\mathit{\mathsf{E}}}}_{\mathit{\mathsf{x}},\mathit{\mathsf{passive}}}}{{\widehat{\mathit{\mathsf{E}}}}_{\mathit{\mathsf{x}},\mathit{\mathsf{passive}}}}\cdot \mathsf{100}\%\kern0.5em . $$

Calculation of changes in power in the SSR is facilitated by the use of ICA for artifact rejection, as EMG signals coming from facial and shoulder muscles would otherwise contaminate the EEG signals, biasing the power in the SSR. Alterations in evoked cortical activation during the active tasks are expressed relative to the passive task by comparing the power in the SSR. The obtained metric is dimensionless, thereby allowing comparison between participants. This metric is also less sensitive to changes in noise (e.g. changes in background cortical activity and EMG activity) due to the voluntary force production.

Laterality indices were calculated for the evoked responses at the electrode level using two sets of electrodes located over the sensorimotor cortices. On the left side of the cortex the following (odd) electrodes were included: F1, F3, F5, FC1, FC3, FC5, C1, C3, C5, CP1, CP3, CP5, P1, P3 and P5. On the right side of the cortex their even counterparts were included: F2, F4, F6, FC2, FC4, FC6, C2, C4, C6, CP2, CP4, CP6, P2, P4 and P6. The electrode sets are referred to as ipsilateral (same side) or contralateral (opposite side) relative to the manipulated wrist. The SNR was averaged for the electrodes on the side contralateral to the disturbance (SNR_contra_) and for the ipsilateral side (SNR_ipsi_), and the sum of both was expressed as ΣSNR. The laterality index for SNR was obtained using:

4 $$ \mathit{\mathsf{L}}\mathit{\mathsf{I}}=\frac{\mathit{\mathsf{SN}}{\mathit{\mathsf{R}}}_{\mathit{\mathsf{contra}}}-\mathit{\mathsf{SN}}{\mathit{\mathsf{R}}}_{\mathit{\mathsf{ipsi}}}}{\mathit{\mathsf{SN}}{\mathit{\mathsf{R}}}_{\mathit{\mathsf{contra}}}+\mathit{\mathsf{SN}}{\mathit{\mathsf{R}}}_{\mathit{\mathsf{ipsi}}}}, $$

which is similar to lateralization indices previously obtained in for example fMRI [34] and EEG [35]. The laterality index is bounded between -1 and 1, where 1 indicates only contralateral activity and -1 indicates only ipsilateral activity.

### Statistical analysis

Statistical analysis on the outcome metrics SNR and LI was performed using a one-way analysis of variance (ANOVA) over the different sensory impairment groups (severe, mild, none, and control). Post hoc analysis using Tukey’s honest significant difference criterion was performed if a significant difference between groups was observed. Statistical analysis on the outcome metric ΔE was performed within each group using a Wilcoxon signed rank test. All tests were performed using a two-tailed significance level of 95% (α = 0.05).

#### Relation between EEG-derived outcome metrics and estimation of anatomical damage

##### Anatomical damage

The structural magnetic resonance images were analyzed to estimate the volume of the sensory and motor tracts (SMT) affected by the stroke lesion. A participant-specific lesion mask was created from the T1-weighted volumes using the LINDA toolbox for automatic segmentation of chronic stroke lesions [36]. The volume of the SMT affected by the stroke lesion was estimated by comparing the person-specific lesion mask against the mask *corticospinal tract* obtained from the John Hopkins University white-matter tractography atlas [37] included in the FMRIB Software Library [38]. Noteworthy, this mask incorporates both descending and ascending fibers. To validate the SMT lesion volume as a metric of sensory impairment, the rank correlation between EmNSA-UE and SMT lesion volume was computed.

##### Regression analysis

Linear regression was used as a means to evaluate the relationship between the EEG-derived outcome metrics and sensory impairment. LASSO regression [39] was used to fit a linear model from the outcome metrics to the SMT lesion volume, using ten-fold cross-validation. The LASSO regression improves the generalization of the linear model (via shrinkage) and can help determining the importance of the predictor variables. The evaluation was conducted for the passive and active tasks separately, with an additional model combining the outcome metrics of both tasks. The performance of the linear model was evaluated using the variance-accounted-for (VAF). Statistical significance was determined by comparing the model performance against data generated using 1000 permutations of the SMT lesion volume.

## Results

The average torque in the passive task was expected to be close to 0 Nm, which could be altered due to passive wrist stiffness when the wrist was placed in the neutral position. Only one participant (ID 8, individual with mild sensory impairment) demonstrated a substantial (>0.3 Nm) passive wrist torque of 2.9 Nm. Due to this large torque under passive conditions, this participant (who only performed the passive task) was excluded from further analysis, as such alteration results in a different task execution. All other participants performed the passive task without substantial wrist torque and without significant increases in EMG activity on wrist flexor and extensor (paired *t*-test between wrist EMG during passive task and rest: relaxed wrist without robotic manipulation). Participants who successfully performed the active task had a high percentage of periods which fulfilled the task criteria: 93% (SD = 8) for the severe group, 95% (SD = 12) for the mild group, 98% (SD = 3) for the no impairment group and 93% (SD = 11) for the control group.

### Signal-to-noise ratio and laterality index

**Fig. 2:**
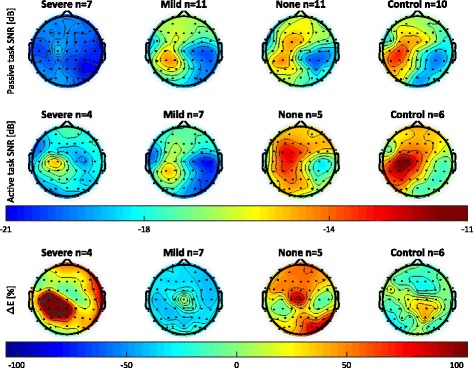
Average SNR and change in power in the SSR for the different sensory impairment groups. The number of participants in a group is indicated by *n*. The results for all recordings performed on the left hand were flipped with respect to the sagittal plane, such that left in these topographic representations is always contralateral to the perturbation. Topographic representations of SNR reveal that: (i) in the passive task the group with severe sensory impairment has a reduced evoked response as compared to all other groups, (ii) in the active task both the severe and mild sensory impairment groups demonstrate a reduced evoked response as compared to the no sensory impairment group and the control participants, and (iii) all the observed response occur around the contralateral sensorimotor cortices. Topographic representation of ∆E reveals an overall decrease of power in the evoked response for the group with mild sensory impairment

**Fig. 3:**
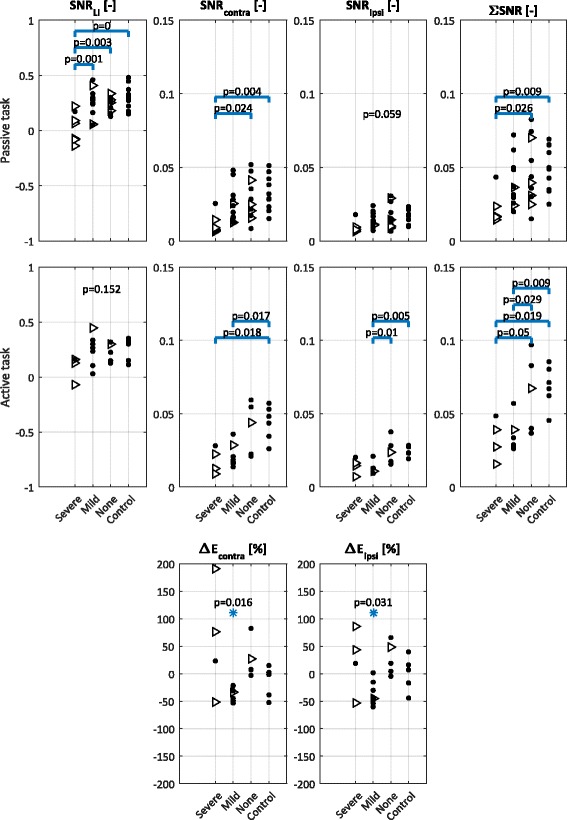
Outcome metrics and statistical analyses. Top and middle row: laterality index for SNR, SNR over contralateral (SNR_contra_) and ipsilateral (SNR_ipsi_) sensorimotor cortices and the total SNR (ΣSNR) for the passive and active task respectively. Horizontal bars (in blue) indicate significant differences between groups; in case there was no significant difference between groups, the p-value of the ANOVA is reported Bottom row: change in power in the SSR in the active task as compared to the passive task (ΔE). The left graph is the ΔE for the contralateral hemisphere and the right graph is the ΔE for the ipsilateral hemisphere. Asterisks indicate a median power change significantly different from zero. Triangles indicate participants with FMA-UE score lower than 40 (i.e. with severe motor impairment), and dots indicate participants with higher FMA-UE scores (i.e. with mild or no motor impairment). The statistical analysis shows that most outcome metrics obtained from the passive task significantly differ for the group with severe sensory impairment. For the active task, the laterality index does not differ over groups; the other parameters indicate reduced responses for the severe and mild sensory impairment groups

Figure 2 shows the SNR for each electrode averaged across the participants in each group and Fig. 3 shows the individual results. As expected, in the passive task (Fig. 2, top row) the control group demonstrates the highest SNR over the contralateral sensorimotor cortex (left side in Fig. 2). The groups of participants with mild and no sensory impairment also show the highest SNR over the contralateral sensorimotor cortex. The lateralization of the SNR is not observed in the group of participants with severe sensory impairment. Moreover, the SNR is low in the severe sensory impairment group compared to the other groups, indicating that sensory input does not reach the scalp electrodes. The laterality index for the passive task (Fig. 3, top row, most left graph) quantifies the differences seen in the scalp maps and shows a significantly altered laterality index for the severe sensory impairment group. The laterality index of close to zero indicates equal contributions from the contra- and ipsilateral cortices. The other graphs in Fig. 3 illustrate that this group has a significant reduction in SNR on both sides of the cortex. One participant in the severe sensory impairment group (ID 7) demonstrated a markedly higher SNR and FMA-UE score in comparison to other participants in this group. This participant had problems with concentration during EmNSA-UE, which could have interfered with the clinical assessment resulting in a low EmNSA-UE score as opposed to actual sensory impairment.

In the active task (Fig. 2, middle row), the scalp distribution of the average SNR for the different sensory impairment groups show higher SNR for the control and no impairment groups than the mild and severe impairment groups. Once again, these differences are quantified by the laterality index (see Fig. 3, middle row, most left graph). The laterality index is positive for the control and no sensory impairment groups. The laterality index for the severe and mild sensory impairment groups still presents positive values, but it also includes participants with a laterality index close to zero. The low SNR for participants with severe and mild sensory impairment is evident in the components of the laterality index (contralateral, ipsilateral, and total SNR).

### Power change

The bottom row of Fig. 3 shows the percentage power change (ΔE) in the SSR in the active compared to the passive task. The active task results for the mild sensory impairment group not only demonstrate a lower SNR than the unimpaired participants and participants with stroke without sensory impairment as concluded from Fig. 3 (middle row), these participants also have a negative ΔE. The negative ΔE indicates that the reduced SNR is not (solely) due to an increase in “noise” in the active task, but that the “signal” (e.g. SSR) is reduced. The ΔE for the severe sensory impairment group has a high variance across participants. This can be explained by the total absence of an SSR in the passive task and a minor SSR in the active task, causing the percentage change to be very large. The active task was only performed if allowed by time and stamina. Other reasons for not performing the active task included complications with the experimental setup in setting the correct force level.

### Evaluation of sensory and motor tract integrity

The rank correlation between EmNSA-UE and SMT lesion volume indicates that lower EmNSA-UE scores are associated with larger SMT lesion volumes (Spearman’s ρ = -0.5, p = 0.005), thus enabling the use of SMT lesion volume as supporting anatomical evidence for sensory impairment.

The linear model for estimation of SMT lesion volume based on EEG-derived outcome metrics obtained from the passive task (SNR_contra_, SNR_ipsi_, and LI_SNR_) explained 75% of the variance in the actual SMT lesion volume (p < 0.01, *n* = 29), where the algorithm indicated that all outcome metrics contributed to this model. This finding indicates a relation between the proposed EEG outcome metrics and the anatomical damage to the SMT. To further investigate the relevance of these outcome metrics in the active task, several steps were performed. The model was again estimated using the outcome metrics from the passive task, yet only for the participants who performed the active task. This resulted in a similar model performance of 77% (p < 0.01, *n* = 16). Interestingly, when using the outcome metrics (SNR_contra_, SNR_ipsi_, and LI_SNR_) obtained from the active task, the model performance decreased to 45% (p < 0.05, *n* = 16). Additionally, a model including outcome metrics from both tasks explained 75% of the variance (p < 0.01, *n* = 16), which does not represent an improvement over using the outcome metrics from only the passive task exclusively.

## Discussion

The goal of this study was to quantitatively assess the integrity of the somatosensory system in individuals with chronic hemiparetic stroke using a combination of robotic continuous joint manipulation and high-density EEG. Continuous wrist manipulation under *passive* conditions results in contralateral cortical evoked responses in unimpaired participants and participants with chronic stroke with mild and no sensory impairment. In contrast, in participants with chronic stroke and severe sensory impairment the evoked responses are strongly reduced or absent in both ipsi- and contralesional sides of the brain and thus not lateralized to either hemisphere. Under *active* conditions, participants with mild sensory impairment show a reduction in power of the cortical evoked responses in both hemispheres, as compared to the passive condition, whereas unimpaired age-matched participants and participants with no sensory impairment do not show this reduction.

### Cortical activation in the passive task

The distribution of SNR over the scalp of unimpaired participants covered electrode sites overlaying the contralateral primary somatosensory cortex. Continuous joint manipulation provokes the flow of proprioceptive and tactile sensory information to the cerebral cortex. This information is mainly mediated by the dorsal column-medial lemniscal pathway, which connects the mechanoreceptors in the periphery to the contralateral primary somatosensory cortex via the ventral posterolateral nucleus of the thalamus. From the primary somatosensory cortex, the somatosensory information is distributed mainly to the secondary somatosensory and posterior parietal cortices [21, 40, 41]. The results of this study are consistent with the distribution of cortical areas listed above, and are similar to cortical activation patterns previously reported for mechanically evoked SSR [14, 42, 43].

In the current study, participants with chronic stroke and mild and no sensory impairment do not demonstrate a significantly altered SNR (both contra- or ipsilateral) as compared to unimpaired participants. In contrast, participants with severe sensory impairment show a lower contralateral SNR in comparison with the other groups. This finding is in line with previous research in individuals with stroke and sensory impairment, in which the cortical responses to median nerve stimulation were reported to be severely decreased or absent (see [44]). A recent study showed that the cortical responses to joint manipulation are reduced in individuals with subacute stroke and motor impairment [45]. The lower SNR suggests altered connectivity between the periphery and the contralateral sensorimotor cortices, as a result of stroke-induced damage along the sensory and motor tracts. As demonstrated in the participants with stroke included in the current study, the diminished cortical responses to a sensory stimulus seem to be related to the level of sensory impairment, which does not necessarily correspond to the level of motor impairment (see Table 1). In general, sensory function can be unimpaired while there might be severe motor impairment, depending on which neural tracts are affected [46]. As human movement control requires sensorimotor integration, it requires functioning of both the afferent and efferent pathways. Damage to either pathway will affect motor control.

Besides lower contralateral responses, participants with severe sensory impairment also exhibit lower ipsilateral responses, in comparison to unimpaired participants. The combination of reduced contralateral and ipsilateral responses causes the laterality index to shift towards zero, i.e. no lateralization of the response. Previous neuroimaging studies using a laterality index to assess cortical activity during hand movement reported a shift in the laterality index (i.e. closer to zero or negative) associated with an increased recruitment of ipsilateral (i.e. contralesional) cortical brain areas [17–20], possibly via corticobulbospinal pathways [47]. Such lateralization is likely related to changes in voluntary motor drive instead of sensory afferents. Campfens, et al. [45] reported, in individuals with subacute stroke, lateralization of cortical evoked responses towards the ipsilateral hemisphere (relative to the stimulated arm). This shift was interpreted as increased responses of the ipsilateral cortex (i.e. contralesional), without reporting the actual metrics for the responses of the ipsilateral cortex. Ipsilateral evoked responses to continuous joint manipulation could be mediated by transcallosal or thalamic pathways [21, 48, 49]. Although transcallosal pathways can transfer information from the contralateral (i.e. ipsilesional) to the ipsilateral (i.e. contralesional) hemisphere this requires information arrives first at the contralateral sensorimotor cortex. Moreover, there is no evidence of thalamic pathways connecting mechanoreceptors in the periphery to the ipsilateral sensorimotor cortices. In general, increased ipsilateral activation could be the result of reduced interhemispheric inhibition, potentially allowing information from the periphery to reach the ipsilateral somatosensory cortices. Interhemispheric inhibition is drastically altered in individuals who underwent a hemispherectomy; in these individuals ipsilateral activation of somatosensory cortices is sometimes observed in response to sensory stimulation [50, 51]. However, the hemispherectomy was often performed at a young age and these individuals were studied many years after surgery, resulting in a long time span during which brain plasticity can occur. In the current study there is no evidence for an increase of ipsilateral evoked responses to joint manipulation.

The group with severe sensory impairment consisted of participants with both proprioceptive and tactile impairment, as established from the EmNSA-UE. The mild sensory impairment group consisted of participants with only tactile impairment. Interestingly, in this study there were no participants with chronic stroke who only had proprioceptive impairment but no tactile impairment. The recorded cortical evoked responses are generated by mechanoreceptors, however in the current approach it is not possible to distinguish between contributions from proprioceptive and tactile sensors. Inclusion of participants with stroke and only proprioceptive impairment would allow for further investigation of the corresponding sources of the cortical evoked response. Previous research by Mima et al. [13] established that under passive conditions the cortical evoked responses due to finger joint manipulation are mainly due to proprioceptive and not tactile sensors. This is in line with the current finding that the lowest SNR under passive conditions was obtained for participants with proprioceptive impairment. The nature of the responses under active conditions might be altered as information about pressure on the hand (i.e. obtained by tactile sensors) would aid task execution when the objective is to maintain a certain force level.

### Alterations to cortical activation during the active task

In the current study, participants with mild sensory impairment show a significant decrease of SSR power in both the contra- and ipsilateral hemispheres during the active task, as compared to the passive task. For participants with severe sensory impairment, the change in power in the SSR could not be accurately determined due to the absence of responses in the passive task. However, during the active task, participants with severe sensory impairment show an equally low SNR as compared to the participants with mild sensory impairment. Thus, the groups of participants with mild and severe sensory impairment demonstrate lower cortical evoked responses during the active task, as compared to unimpaired participants and participants with stroke without sensory impairment. In unimpaired participants and participants with no sensory impairment no significant differences were observed between passive and active conditions. The latter finding is in agreement with previous studies. Cortical activity during a wrist flexion task with joint manipulation was performed in individuals with stroke in two other studies with transient [52] and continuous [45] joint disturbances. Both studies reported metrics based on evoked responses for passive and active conditions, with small differences between conditions. Interestingly, in these studies the active condition was only performed by individuals with stroke and FMA-UE scores above 40 points without any sensory impairment.

The decreased evoked responses in participants with mild sensory impairment suggest a reduction in sensory information relayed to the brain, either due to reduced sensory signals from the periphery, changes in the mechanisms of sensorimotor integration related to sensory impairment, or both. Importantly, the disturbance signal applied by the robotic manipulator was the same under passive and active conditions. In the peripheral nervous system, the active wrist flexion causes the proprioceptive and tactile sensors to operate in a different range in comparison to the passive task, as the proprioceptors are shortened due to the muscle contraction (and lengthened for the antagonist muscle) and the tactile sensors on the hand register the increase pressure applied to the handle. In response, the central nervous system might modulate the sensitivity of the muscle spindles using alpha-gamma motor neuron co-activation, to compensate for the changing afferent signals [53]. Impaired sensory function may lead to impaired feedback control, which could affect the modulation of muscle spindle and gamma motor neuron co-activation. Additionally, active movement induces changes in the activity of the sensorimotor cortices observed as suppression of the mu and beta rhythms during preparation and execution of movement [54]. A similar phenomena occurs during passive movements [55–57], suggesting that suppression of cortical rhythms is partly related to neural processing of sensory input. In individuals with motor impairments after stroke, suppression of the beta rhythm is significantly reduced during active movement [58] and following somatosensory stimulation (passive movement and tactile stimulation) [56, 59]. These alterations are related to changes in excitation and inhibition through varying levels of γ-aminobutyric acid (GABA) [58], which are considered detrimental for motor control and could be linked to the observed decrease of the evoked responses. Extra insight into the source of the reduced responses could be obtained by directly measuring the output of the sensors in the periphery, for example using microneurography [60], and by measuring the induced changes to cortical mu and beta rhythms as metrics of impaired sensorimotor integration [59].

Ipsilateral cortical activity during voluntary motor drive has been shown before in individuals with chronic stroke and severe motor impairments [17–20]. Here, the focus is on quantifying the cortical responses evoked by continuous joint manipulation (sensory information) and determining if there is lateralization of sensory information to the ipsilateral hemisphere. The results in this study do not show a consistent shift of cortical evoked responses towards the ipsilateral hemisphere for any group. This result suggests that proprioceptive and tactile information is transmitted to the contralateral hemisphere only, in accordance with known anatomic constraints (i.e. dorsal columns). This lack of sensory information reaching the contralateral and the ipsilateral cortex is likely to hamper any role of the ipsilateral cortex in feedback control (e.g. reflex modulation).

### Robotic joint manipulation to assist the assessment of sensory impairment

Continuous joint manipulation allows studying somatosensory cortical evoked responses during the execution of meaningful control tasks. With this approach it is possible to measure the SNR and SSR to quantitatively assess the integrity of the sensory pathways under passive and active conditions, while being certain of stimulating the sensory systems involved in movement control (i.e. proprioceptive and tactile). Determining the integrity of sensory pathways in the context of movement control provides additional information for the accurate description of sensory impairment of a stroke patient. This information can assist the development and selection of patient-specific rehabilitation programs (and interventions) that promote plastic reorganization of the remaining cerebral networks [24, 61], with the ultimate goal to improve functional outcome.

Current clinical practice determines sensory and motor impairment based on subjective expert evaluation using established clinical assessments, which are vulnerable to issues related to validity and reliability [62]. The majority of the existing clinical assessments focus on describing motor-related impairments such as weakness, spasticity, and pathological synergies (e.g. [22, 63, 64]), even though the assessment of sensory impairment is necessary for proper selection and evaluation of stroke rehabilitation interventions [5–7, 65]. Alternatively, methods for objective quantification of brain function rely on neuroimaging techniques [66]. When using indirect, blood oxygenation level dependent neuroimaging techniques, the poor temporal resolution hampers studying the cortical evoked response, which in turn hinders any distinction between cortical activation due to sensory information processing or voluntary motor drive. A known way of quantitatively assessing *sensory* function of the upper limb using neuroimaging techniques is the characterization of somatosensory evoked responses to electrical stimulation of the median nerve. Sensory function is then described based on the latency of the peaks in the cortical evoked response as measured using MEG or EEG [67]. However, by applying an electrical stimulus one cannot be sure which sensory system is actually being stimulated, as there are many types of afferent fibers (e.g. for conveying pain, temperature, tactile, or proprioceptive information). Furthermore, electrical stimulation is generally applied under passive conditions and provides a non-physiological type of activation of sensory nerves. Because control of human movement demands ongoing sensorimotor integration, it is desirable to evaluate the status of the sensory system while engaged in a meaningful sensorimotor task.

In this study, the relation between the EEG-derived outcome metrics and the integrity of sensory and motor tracts is demonstrated by the successful estimation of SMT lesion volume by a linear regression model obtained from the outcome metrics measured from the passive task. Adding the outcome metrics measured from the active task did not improve the model performance. This finding emphasizes the importance of the passive task for revealing the integrity of the connections between the periphery and sensorimotor cortices. The information obtained from the active task likely reflects other aspects of the sensorimotor system, for example altered sensorimotor integration.

This study demonstrates the quantitative assessment of the integrity of the somatosensory system through continuous joint manipulation. Although the passive and active task require some capabilities of the participants in terms of cognition and ability to sit upright independently, the protocol is feasible for individuals with chronic stroke, even in the presence of severe motor impairment. The passive task could be executed by all participants in this study. The active task could be executed by most participants, except for participants lacking voluntary wrist flexion (*n* = 3), which is related to severe motor impairment. In the current study, participants with FMA-UE scores of nine and lower were incapable of voluntarily flexing their wrist.

### Limitations and future directions

Previous work by Vlaar et al. [14, 68] revealed that the relation between continuous wrist manipulation and cortical evoked responses is highly nonlinear, yet the responses are periodic with the disturbance signal. The implication is that a linear metric will not be able to capture the relationship between disturbance at the wrist and cortical responses. Although the metrics in the current study do not attempt to describe this relationship, they can successfully quantify the full periodic response (i.e. both linear and nonlinear contributions). The repeatability of these metrics has yet to be verified; however, test-retest reliability of mechanically evoked steady-state responses has previously been established. Pang and Mueller [69] demonstrated that the amplitude of the evoked cortical response does not vary over recording sessions in unimpaired young participants using continuous tactile stimulation.

Due to the specific focus on the wrist in this study, some elements of the EmNSA could have been omitted (e.g. tactile sensation of the upper arm and proprioception of the shoulder and elbow). However, omitting these scores would not alter the way participants are classified. Two participants (ID 2 and 5) would receive a 0 instead of a 1 for proprioception, but both would still remain in the severe sensory impairment group. Indeed, sensory impairment is highly correlated between segments of a limb for the same sensory modality [70].

To further develop relevant outcome metrics for sensory impairment, it would be important to relate these outcome metrics to the current golden standard in sensory assessment, i.e. EmNSA. However, this raises several issues, as clinical assessments typically use ordinal scales and no normative data are available. The EmNSA only assesses passive movement and investigates all sensory modalities separately. During (natural) movement control, there is always interplay between sensory modalities. Although the applied wrist joint manipulation stimulates multiple sensory systems at the same time and therewith reduces the ability to distinguish them, the system is assessed in a way reflecting everyday control, making comparison to EmNSA not straightforward. In the current study, sensory impairment was related to SMT integrity as estimated from the location of the stroke lesion. An attractive alternative is the quantification of SMT integrity by means of diffusion tensor imaging [71], which can directly measure the integrity of the sensory and motor tracts.

The metrics demonstrated in this study (SNR, SSR, and the laterality index) may allow for tracking sensory impairment over time, which is of specific interest in the acute and subacute phases of stroke recovery. Most recovery of neurological impairment is spontaneous and takes place in the first six months after stroke [24, 72]. At the end of this period, a significant number of individuals show poor recovery of upper limb function and thus do not follow the proportional recovery rule [73, 74], which predicts that individuals with stroke will recover approximately 70% of the difference between the maximum FMA-UE score and their initial score. Although the underlying cause of poor recovery is not understood, recent studies indicate that early assessment of corticospinal tract integrity has the potential to identify individuals with poor recovery [75, 76]. Importantly, individuals with poor recovery of upper limb function also present impairments such as aphasia [77] and visuospatial neglect (when affected in the same brain hemisphere) [78]. Thus, poor recovery after stroke may be linked to a multimodal suppression of brain function, which possibly also includes sensory function. Quantitative outcome metrics obtained from longitudinally monitoring sensory impairment, starting very early after stroke onset, allows investigating the effects of therapy on the recovery after stroke and the potential use of these metrics as neurophysiological biomarkers of recovery that may predict final outcome post stroke [79]. This latter aim is in line with previous worldwide initiatives to achieve consensus in stroke recovery research [80] and prognostic modeling [81].

### Added value

This study demonstrates an approach to quantitatively assess the integrity of the somatosensory system using EEG and a robotic manipulator that applies periodic disturbances to the wrist joint. This setup allows for analysis of the evoked cortical responses to robotic joint manipulation in individuals with stroke during upper limb control. The advantage of this approach is that it specifically stimulates sensory systems involved in movement control, in contrast to electrical stimulation. The evoked responses can be studied during a passive condition, revealing connectivity between the periphery and the cortex. Additionally, studying the evoked responses under active conditions allows insight in alterations due to engagement of the sensorimotor system in a meaningful movement control task.

## Conclusions

- Using the electroencephalogram and a robotic manipulator allows for quantitative assessment of evoked cortical activity reflecting proprioceptive and tactile information during meaningful upper limb control tasks executed under both passive and active conditions.
- In individuals with mild and no sensory impairment in the chronic phase of stroke, the cortical representation of somatosensory stimuli of the affected upper limb is lateralized to the contralateral hemisphere, as seen in age-matched unimpaired individuals.
- The cortical representation of somatosensory stimuli is not lateralized to the contralateral hemisphere in individuals with severe sensory impairment in the chronic phase after stroke. The absence in lateralization results from a reduction in responses in the contralateral hemisphere and not by an increase in responses in the ipsilateral hemisphere.
- Individuals with mild sensory impairment after stroke have reduced cortical representation of somatosensory stimuli under active conditions as compared to passive conditions. This reduction does not occur in unimpaired individuals and individuals without sensory impairment after stroke.

## Abbreviations

ANOVA: Analysis of variance
EEG: Electroencephalogram
EmNSA-UE: Erasmus modification of the Nottingham Sensory Assessment of the upper extremity
FMA-UE: Fugl-Meyer assessment of the upper extremity
ICA: Independent component analysis
LI: Laterality index
MVC: Maximum voluntary contraction
SMT: Sensory and motor tracts
SNR: Signal-to-noise ratio
SSR: Steady-state response
